# Case Report: A rare case of bone destructive sacrococcygeal chordoma presenting as anal distension

**DOI:** 10.3389/fonc.2025.1672249

**Published:** 2025-12-10

**Authors:** Xianshui Mei, Ming Li, Kun Tang, Ran Tang, Xudong Wang, Ping Cheng, Li Chen, Afen Wu, Jiuxiang Wang, Jianming Wang

**Affiliations:** 1 Department of Anorectal Surgery, The First Affiliated Hospital of Anhui University of Chinese Medicine, Hefei, Anhui, China; 2 School of Pharmacy, The First Affiliated Hospital of Anhui University of Chinese Medicine, Hefei, Anhui, China; 3 Department of Pathology, The First Affiliated Hospital of Anhui University of Chinese Medicine, Hefei, Anhui, China; 4 Experimental Center of Clinical Research, The First Affiliated Hospital of Anhui University of Chinese Medicine, Hefei, Anhui, China

**Keywords:** sacrococcygeal chordoma, anal distension, sacral nerve, radiotherapy, surgery

## Abstract

Various pathologies, environmental factors, and medications can cause anal distension. Sacrococcygeal chordoma is a rare tumor originating from the remnants of the primitive spinal cord. Nonetheless, only a few case reports of anal swelling caused by sacrococcygeal chordoma have been published. This article reports a female patient with a history of mastectomy due to breast cancer who presented with anal swelling and was finally diagnosed with sacrococcygeal chordoma. The patient experienced anal swelling and discomfort that worsened at night. A smooth, oval-shaped mass was palpable in the rectum, 4 cm from the anal verge near the coccyx. The mass was tender and no blood was found on the glove after examination. A mass shadow was observed in and around the S2-5 vertebrae according to MRI. Immunohistochemical analysis revealed strong positivity for vimentin(VIM), cytokeratin(CK) AE1/AE3, epithelial, embrane antigen(EMA), and S-100 protein. The cytogenetic marker SMARCB1/INI1 was positive but not missing, and the Ki-67 proliferation index was approximately 5%. These findings confirmed the diagnosis of sacrococcygeal chordoma. This article aims to expand the knowledge about sacrococcygeal chordoma and its pathogenesis. Notably, chordoma can exclusively cause anal swelling, and clinicians should consider differential diagnosis in practice.

## Introduction

Anal distension is a conscious symptom caused by local irritation of anorectal lesions and can be caused by various pathologies, environmental factors, and medications (1–3). Therefore, carefully examining and identifying the underlying cause is essential for diagnosis. The sacral nerve is a component of the pelvic plexus nerve, with its lowermost part running along the S4 and S5 vertebrae. Compression of the sacral nerve along the S4 and S5 vertebrae by space-occupying lesions can cause swelling of the anal area (4, 5). Compression mostly arises from pelvic masses, pelvic floor hernia, uterine posterior position, rectal endometriosis, and sacrococcygeal tumor. This article presents a rare case of anorectal swelling resulting from a chordoma that compressed the sacral nerve.

Chordoma is a rare and slow-progression cancer that is thought to originate from residual fetal notochord tissue (6). It predominantly occurs in a disseminated form and has a prevalence of 0.1 per 100,000 (7, 8). Chordomas mainly occur at the base of the skull, mobile spine, and sacrococcygeal region, following the anatomical distribution of the spinal cord. However, cutaneous involvement has also been reported (8–10). The onset of chordoma is insidious and involves no typical clinical symptoms (11). As the disease progresses, chordomas may affect bowel, bladder, and sexual function (12). Other clinical symptoms may include arm and leg weakness, pain, tingling, numbness, deep localized pain, or radiculopathy (6, 13, 14). Notably, few articles reported sacrococcygeal chordoma presenting with perineal and anal distension and discomfort as the primary clinical symptom. This paper describes the case of a 58-year-old female who had undergone a mastectomy for breast cancer. Sacrococcygeal chordoma was diagnosed after the patient reported experiencing perineal and anal distension and discomfort, particularly at night. This article presents a thorough review of sacrococcygeal chordoma, covering its clinical presentation, histopathology, imaging findings, and treatment options. The objective is to enhance comprehension of the disease and its pathogenesis while emphasizing the possibility of anal swelling as a result of sacrococcygeal chordoma.

## Case report

A 58-year-old woman with a surgical history of hysterectomy 16 years prior and mastectomy 6 years ago for breast cancer was referred to our hospital due to anal distension for more than 5 months. At the time of the visit, the patient was still taking anastrozole 1mg once daily (qd) and undergone routine whole-abdomen CT scans and tumor marker tests, all of which revealed no significant abnormalities. As the disease progressed, the perineum also became swollen and painful. Over the past two months, the patient reported increased discomfort at night when sleeping, which was alleviated by standing up. The patient denied any history of dizziness, headache, fever, renal itching, panic attacks, chest tightness, nausea or vomiting. An electronic colonoscopy was performed four months prior at a local hospital, which suggested proctitis. However, the treatment was found to be ineffective. A defecography was performed 20 days ago at another hospital, which revealed anterior proptosis of the anterior wall of the rectum. The patient visited several hospitals during this period and was diagnosed with mixed hemorrhoids, anterior rectal protrusion, and rectal mucosal prolapse.

**Figure 1:**
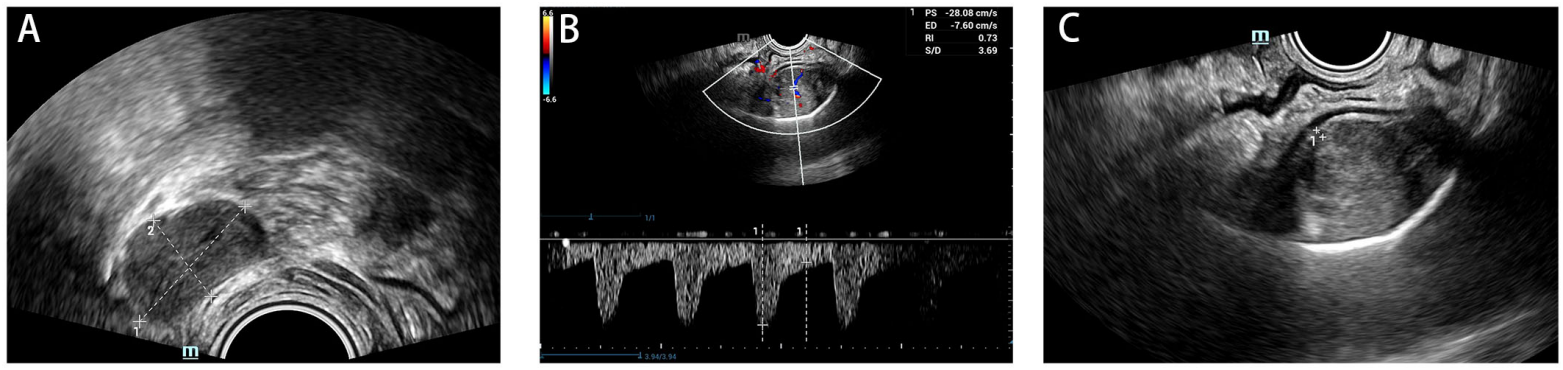
Two-dimensional ultrasonography at the time of diagnosis. A 51mm x 30mm x 71mm hypoechoic mass with heterogeneous internal echogenicity **(A)** striated blood flow information **(B)** was detected posteriorly in the rectum **(C)**.

**Figure 2:**
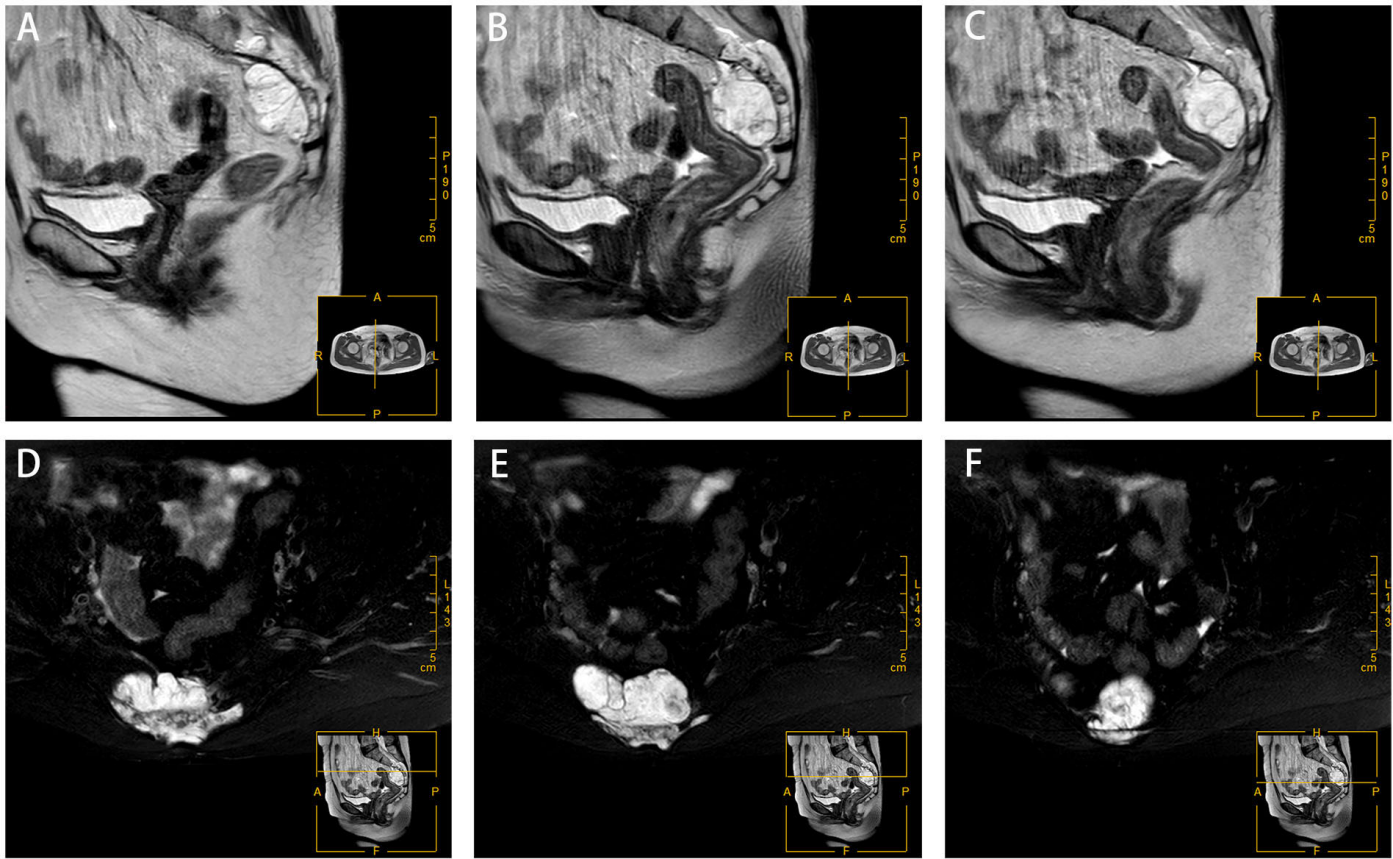
Magnetic resonance imaging at the time of diagnosis. Clumpy long T1 **(A-C)** and long T2 **(C-E)** signals are seen in and around the S2-5 vertebrae.

The patient reported regular bowel movements 1-2 times per day, with no bleeding after defecation. On palpation, a 3cm x 4cm oval mass was found 4cm from the distal margin of the rectum near the tailbone. The mass was firm, smooth, and tender on pressure, with no blood staining on the glove. Further imaging was conducted for diagnosis. Two-dimensional ultrasonography revealed a solid mass measuring 51mm x 30mm x 71mm in the posterior rectum (**Figure 1**). The MRI revealed a visible mass shadow in and around the S2-5 vertebrae. The mass was blood-rich and a few areas of liquefaction were observed internally. Furthermore, sacral bony changes were found in the adjacent sacrum, affecting the S3-S4 anterior margin of the putamen (**Figure 2**). In a tumor marker test conducted at our hospital, the CYFRA21-1 (cytokeratin 19 fragment) value was slightly elevated at 3.32 ng/ml, with the normal value being <3.30 ng/ml. No significant changes were observed in other regular examinations.

**Figure 3:**
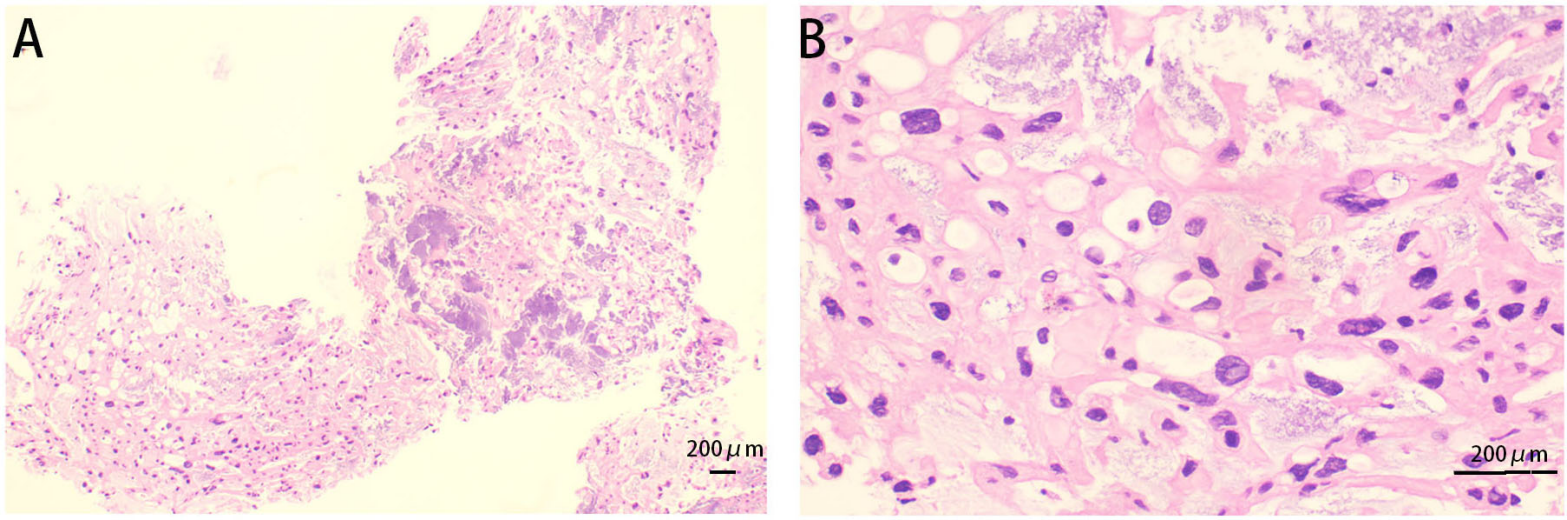
Cystic tissue with HE staining. **(A)** The cells were arranged in cords and small patches (original magnification, x100); **(B)** Cytoplasmic, eosinophilic, or epithelioid cells with translucent, vacuolated, enlarged, and deeply stained nuclei. (Original magnification x 400).

**Figure 4:**
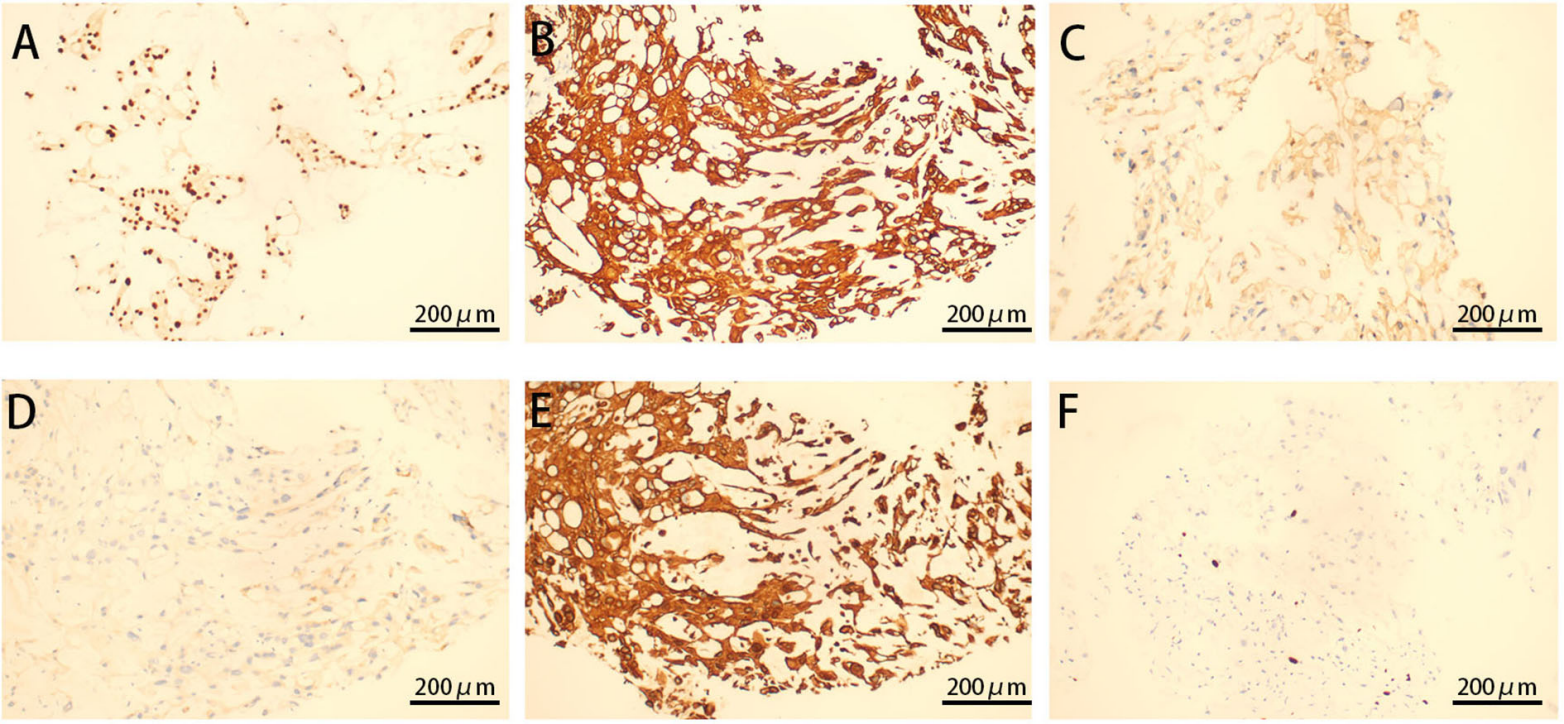
Immunohistochemistry of cystic tissue. Diffusely intense immunostaining for brachyury **(A)**, CK **(B)**, S100 **(C)**, EMA **(D)**, VIM **(E)**, and ki67 **(F)** expression is displayed (original magnification ×200).

For further diagnosis, a tissue puncture was performed for pathological examination. The HE staining of the punctured tissue revealed cells arranged in stripes and small sheets, with fibrous segregation observed in small lesions. Epithelioid cells showed abundant and eosinophilic cytoplasm, some of which were translucent and vacuolated. Most of the cells had small nuclei, but some of them had enlarged nuclei and deeply stained nuclei (**Figure 3**). The immunohistochemical analysis revealed strong positivity for vimentin, cytokeratin AE1/AE3, epithelial membrane antigen, and S-100 protein (**Figure 4**). The cytogenetic marker SMARCB1/INI1 was positive but not missing (**Figure 4**). The Ki-67 proliferation index was approximately 5% (**Figure 4**). The patient was diagnosed with sacrococcygeal chordoma following a comprehensive pathological examination.

**Figure 5:**
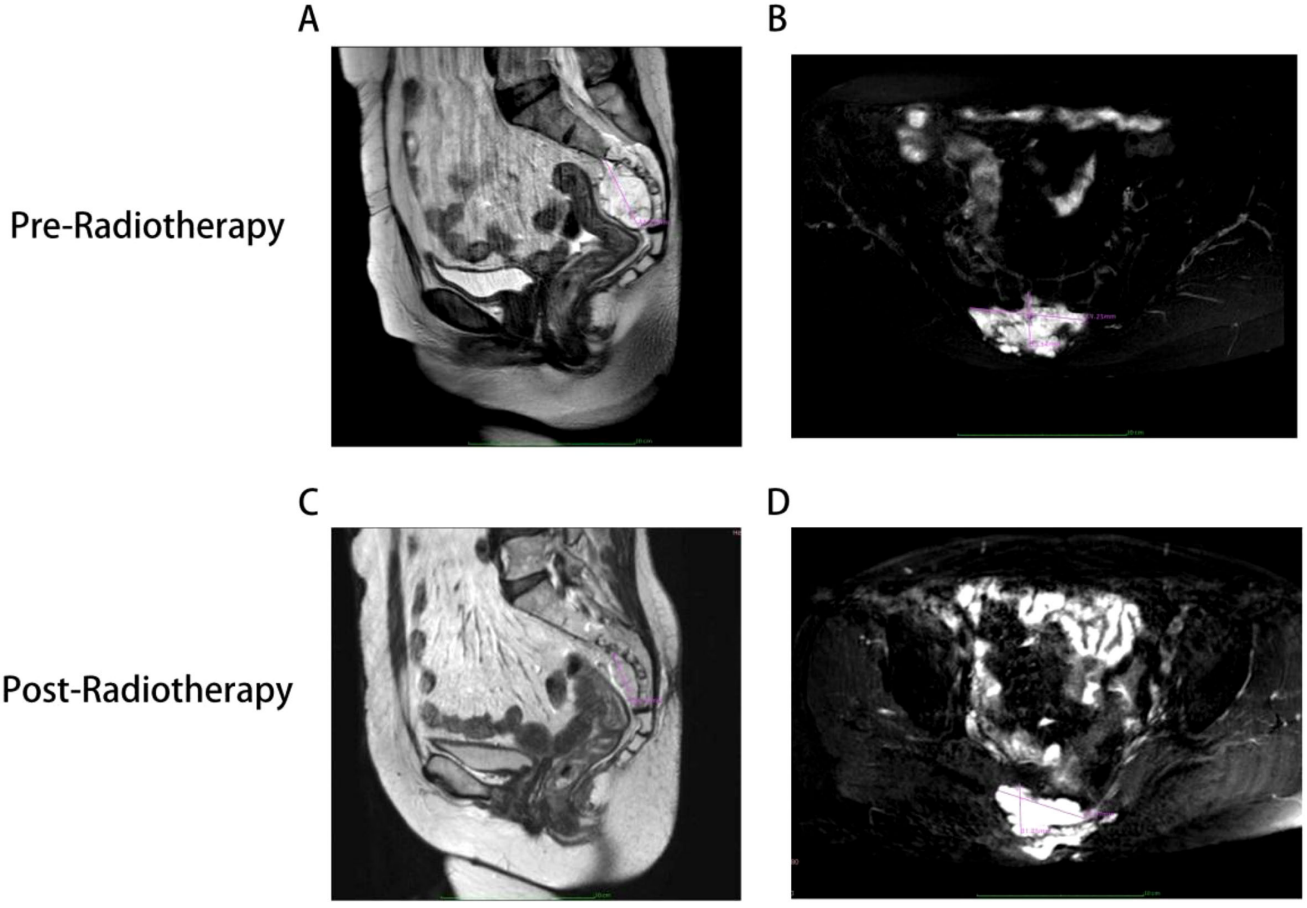
MR findings of the patient before and after radiotherapy. **(A, B)** Show the tumor size of the patient before radiotherapy (4.4cm x 6.9cm x 3.3cm). **(C, D)** Show the tumor size 18 months after the completion of radiotherapy (3.7cm x 5.5cm x 3.1cm). Mass shadow is clearly visible in and around the S2-S5 vertebral bodies, with a distinct Clumpy long T1 **(A, C)** and long T2 **(B, D)** signals.

Owing to the chordoma’s location from S2 to S5, which is associated with a high surgical risk and the potential for multiple complications, the patient ultimately chose to undergo radiotherapy alone without surgery after considering the diagnostic and therapeutic recommendations from several doctors as well as her own family and financial situation. **Figure 5** illustrates the 18-month follow-up results after the patient completed a total of 25 radiotherapy sessions at a dose of 50 Gy, administered five times per week. At the 3-month follow-up after completing radiotherapy, the patient reported a significant reduction in perineal and anal heaviness and pain compared to pre-treatment levels. Although some discomfort persisted, the patient was able to sleep at night. Aside from some erythema and ulceration of the skin at the irradiated site, there were no other discomforts in the rectum or bladder. At the 18-month follow-up after completing radiotherapy, the skin at the irradiated site had healed well. The patient reported that the perineal and anal heaviness had disappeared, and there were no other discomforts.

## Discussion

Anal swelling can be caused by various disorders, including urological, gynecological, psychiatric, and gastrointestinal disorders (2, 15). However, in rare cases, anal swelling can be the primary clinical manifestation of sacrococcygeal chordomas. The patient in this report initially presented with anal swelling, which later progressed to swelling in the perineum. The diagnosis of sacrococcygeal chordoma was made 5 months after the initial clinical symptoms, by which time the patient’s spine had already been damaged (**Figure 1**). Therefore, this article presents a comprehensive account of the patient’s clinical symptoms, the outcomes of various examinations, and the therapeutic program. This report aims to broaden the understanding of the pathological causes of anal swelling and enrich the knowledge about chordoma.

Compression of the sacral nerves in the area of sacral vertebrae 4 and 5 can resulted in anal swelling (16, 17). In this case, the chordoma tissue located in front of the sacrum extended through the sacral foramina of sacral vertebrae 4 and 5 during its growth, potentially directly compressing the sacral nerves of sacral vertebrae 4 and 5, resulting in anal swelling in the patient. As the tumor grew larger, the compression worsened. Meanwhile, as the tumor progressed, the chordoma tissue invaded or compressed other nerves or tissues, causing a feeling of heaviness and swelling in the perineal region. This may explain why the patient initially presented with anal swelling, and as the tumor progressed, swelling and pain appeared in the perineal area. Changes in the patient’s position at night likely exacerbated the compression of surrounding nerves and tissues by the tumor, leading to increased pain at night, and in some cases, pain severe enough to prevent sleep. Pathological examination is essential in diagnosing chordoma (6, 18–21). Ultrasonography revealed the presence of a solid mass in the posterior rectum, while enhanced CT revealed a neoplastic lesion close to the S3-S4 vertebrae. Based on the patient’s history of breast cancer and ongoing anastrozole treatment, the risk of a malignant postoperative breast tumor was considered. The patient’s diagnosis of classic sacrococcygeal chordoma was supported by positive HE staining and cytogenetic markers, including Vim, CK, EMA, cytokeratins, S100 proteins, and SMARCB1/INI1.

The protracted timeline from symptom onset to diagnosis (5 months) is a significant concern. The patient underwent evaluations by several physicians across different hospitals before receiving a definitive diagnosis of chordoma at our institution. Given the patient’s history of breast cancer, regular whole-abdomen CT scans and tumor marker tests were performed, which showed no significant abnormalities. Early presentations of anal fullness led to consultations at other hospitals, where abdominal CT and tumor marker tests were normal, and electronic colonoscopy revealed no significant findings. These results contributed to the misdiagnosis of the symptoms as ordinary anal discomfort, without considering the possibility of extraintestinal tumors. A thorough digital rectal examination (DRE) was not performed diligently. The tumor’s high location, distant from the anal verge, made it difficult to palpate without a meticulous examination. Chordoma, being a rare disease with low incidence, further complicated the diagnostic process. At our institution, the examining physician had a slightly longer index finger, which enabled palpation of the tumor during DRE. This finding prompted further MRI evaluation, leading to the diagnosis. This case underscores the importance of not overly relying on ancillary diagnostic tests and personal experience. Instead, clinicians should re-emphasize the importance of a thorough DRE, especially when symptoms persist despite normal initial investigations.

In conclusion, anal swelling can be caused by various pathological factors. However, anal swelling as the primary clinical symptom of a sacrococcygeal chordoma remains rare. Therefore, anorectal surgeons should consider chordoma as a possible diagnosis in patients presenting with anal swelling. Palpation plays a crucial role in detecting lumbosacral chordoma, as demonstrated in the case (22). The cystic swelling noted on palpation prompted further investigation. The diagnosis and prognosis of chordoma patients can be aided by HE staining and cytogenetic markers, especially brachyury staining of tissues.

## Data Availability

The original contributions presented in the study are included in the article/supplementary material. Further inquiries can be directed to the corresponding authors.
